# Comparative Analysis of *Metopograpsus quadridentatus* (Crustacea: Decapoda: Grapsidae) Mitochondrial Genome Reveals Gene Rearrangement and Phylogeny

**DOI:** 10.3390/ani15081162

**Published:** 2025-04-17

**Authors:** Dan-Dan Bian, Sheng Tang, Song-Nan Wang, Qiu-Ning Liu, Bo-Ping Tang

**Affiliations:** 1 Jiangsu Key Laboratory for Bioresources of Saline Soils, Jiangsu Synthetic Innovation Center for Coastal Bio-Agriculture, Jiangsu Provincial Key Laboratory of Coastal Wetland Bioresources and Environmental Protection, School of Wetlands, Yancheng Teachers University, Yancheng 224007, China; 2 College of Life Sciences, Anhui Agricultural University, Hefei 230036, China; 3 College of Fisheries and Life Science, Shanghai Ocean University, Shanghai 201308, China

**Keywords:** mitochondrial genome, *Metopograpsus quadridentatus*, gene rearrangement, phylogenetic relationships

## Abstract

The taxonomy of the genus *Metopograpsus* is still a matter of debate because its species have limited morphological differences. Mitochondrial genomes, which are highly informative and easily accessible genetic markers, have been widely used to study molecular evolution and clarify relationships among species. This study aims to investigate the mitochondrial genome of *Metopograpsus quadridentatus*, a species with unique ecological and evolutionary significance. By analyzing the mitochondrial genome, we seek to address taxonomic uncertainties and provide insights into the evolutionary history of this species. In this study, we sequenced and analyzed the mitochondrial genome of *M. quadridentatus* to investigate its gene rearrangement patterns and its place within Brachyura. We compared the gene order of *M. quadridentatus* with that of 40 other Brachyuran species and created phylogenetic trees based on the nucleotide and amino acid sequences of 13 protein-coding genes (PCGs). We found that the mitochondrial gene arrangement of *M. quadridentatus* is mostly unchanged, similar to the original crustacean pattern, except for the movement of the *trnH* gene. Notably, the gene orders of several families, such as Eriphiidae, Grapsidae, Camptandriidae, Dotillidae, Plagusiidae, Ocypodidae, and Gecarcinidae, are the same. Phylogenetic analyses consistently placed *M. quadridentatus* within the genus *Metopograpsus* and the family Grapsidae, confirming its current taxonomic classification. These results offer important insights into evolutionary relationships and gene order conservation within Brachyura. Our study highlights the significance of mitochondrial genomes in resolving taxonomic uncertainties within the genus *Metopograpsus*.

## Simple Summary

The marine crab *Metopograpsus quadridentatus* is categorized in the family Grapsidae, but its classification remains unclear because of limited differences in appearance among species. In this research, we examined its mitochondrial genome, which showed a gene order similar to that of ancient crustaceans, with the exception of the translocated *trnH* gene. Analysis of 13 protein-coding genes from 40 Brachyura species indicated that it belongs to the genus *Metopograpsus* and family Grapsidae, clarifying its evolutionary relationships and demonstrating the importance of mitochondrial genomes in taxonomic classification.

## 1. Introduction

Mitochondria are organelles found in eukaryotic cells that house their own genetic material, known as the mitochondrial genome, characterized by a high mutation rate, lack of gene recombination, a high replication rate, and maternal inheritance [1,2]. In metazoans, the mitochondrial genome is a small double-stranded circular molecule typically 16–20 kbp in size [3]. It contains 13 protein-coding genes, including subunits of NADH dehydrogenase, cytochrome b, cytochrome c, and ATP synthase, along with transfer RNA genes, ribosomal RNA genes, and a non-coding control region that regulates the mitochondria genome’s transcription and replication. Mitochondrial DNA (mtDNA) in animals exhibits remarkable organizational precision [4]. It contains 13 protein-coding genes: ND1-6 and ND4L encode core catalytic subunits of Complex I, Cyt b acts as the electron transfer hub of Complex III, COI-III constitute the oxidative center of Complex IV, and ATP6/8 drive proton gradient conversion in Complex V. This compact arrangement, interspersed with 22 tRNA genes, reflects evolutionary optimization for genomic compression and expression regulation. Notably, newly identified regulatory elements within the D-loop control region—such as termination-associated sequences (TASs) and conserved sequence blocks (CSBs)—participate in replication initiation through secondary structure formation, with their polymorphic features now serving as vital molecular markers in population genetics [5].

The infraorder Brachyura consists of 98 families and nearly 7000 described species [6]. *Metopograpsus quadridentatus* is a type of marine crab commonly found in rock crevices or under rocks at low tide. It is found in various locations, including Malacca, Java, Bali, Kalimantan, New Guinea, the Indian Ocean, Guangdong, Fujian, Zhejiang, Shandong, and other areas in China [7]. Currently, *Metopograpsus quadridentatus* is classified under Crustacea, Decapoda, Brachyura, Grapsidae, genus *Metopograpsus*. Due to advancements in sequencing technology, especially next-generation sequencing, the time and cost required for sequencing have substantially decreased, making it simpler to acquire the mitochondrial genome sequence [8]. Mitochondrial gene-based molecular phylogenetic analyses first confirmed the monophyly of all six *Metopograpsus* crab species, revealing high intraspecific genetic variation and a pronounced phylogeographic structure in *M. thukuhar* and *M. quadridentatus*, suggesting the presence of undescribed evolutionarily significant units (ESUs) that require further taxonomic validation via nuclear DNA and morphological investigations [9]. Mitochondrial genome analysis is widely used in phylogenetic analysis, biogeography, population genetics, medicine, and ecology [10,11,12,13,14]. The mitochondrial genome of *Helice wuana* and its relatives offer crucial evidence on the origin, germline evolution, and unique genetic structure of crab [15]. The mitochondrial genome contains 37 genes, which theoretically have a high rearrangement potential. However, according to existing results, gene rearrangements are not very common. Similar gene rearrangements among different species are thought to be unlikely due to convergent evolution but instead suggest a certain phylogenetic relationship among species [16]. As a result, analyzing gene rearrangement is more appropriate for determining the superior phylogenetic relationship [17].

In this research, we analyzed the complete mitochondrial genome of *M. quadridentatus* and compared it with other brachyura species. By examining the amino acid and nucleotide sequences of 13 protein-coding genes, we built phylogenetic trees. Additionally, we studied the gene rearrangement patterns to determine the evolutionary placement of *M. quadridentatus* within the brachyura.

## 2. Materials and Methods

### 2.1. Sampling and DNA Extraction

Mature crabs (*M. quadridentatus*) were gathered from Xiamen, Fujian Province, China. Prior to DNA extraction, the specimen was kept in aerated tap water at a temperature of 21 ± 1 °C for a period of 1 week. DNA was extracted from muscle tissue samples using an Aidlab Genomic DNA Extraction Kit (Aidlab, Beijing, China), and the extracted DNA was then preserved at −20 °C until amplification.

### 2.2. Sequencing and Analysis

Prior to mitochondrial genome amplification of *M. quadridentatus*, commercially prepared universal primers were obtained from Sunbiotec (Beijing, China) [18]. Genomic DNA amplification was conducted following standardized protocols provided with the Aidlab Extraction Kit (Beijing, China). Polymerase chain reactions were executed in 50 μL volumes comprising 5 μL of 10× Taq Plus Buffer (Mg^2+^ supplemented), 4 μL dNTP mixture, 2 μL forward/reverse primers (each), 2 μL template DNA, 34.5 μL molecular-grade ddH_2_O, and 0.5 μL Taq DNA polymerase RED. Thermal cycling parameters included an initial denaturation period at 94 °C for 3 min, followed by 35 cycles of denaturation at 90 °C for 30 s, primer-specific annealing (49–58 °C for 35 s, with temperature optimized per primer pair), and extension at 72 °C (30 s to 4 min duration, scaled by target fragment size), concluding with terminal elongation at 72 °C for 10 min. Electrophoretic separation was conducted using 1% agarose gels (*w*/*v*) for PCR product visualization, followed by purification with Aidlab’s DNA Gel Extraction System (Beijing, China). The processed amplicons were subsequently cloned into T-vector systems for sequencing analysis (Sangon Biotech, Shanghai, China).

### 2.3. Bioinformatics Analysis

Mitochondrial genome annotation of *M. quadridentatus* was performed via NCBI BLAST homology searches (https://blast.ncbi.nlm.nih.gov/Blast.cgi). Sequence assembly and refinement were conducted using DNASTAR’s SeqMan module version 7 (DNAStar Inc., Madison, WI, USA) [19]. Genome architecture was visualized through OrganellarGenomeDRAW (OGDRAW) to generate a graphical genome map [20]. Transfer RNA identification and secondary structure prediction (cloverleaf conformation) were achieved using the tRNAscan-SE algorithm [21]. Nucleotide composition, synonymous codon usage bias, and compositional skewness (AT skew = [A − T]/[A + T]; GC skew = [G − C]/[G + C]) were quantified using MEGA 12 [22,23].

### 2.4. Phylogenetic Analysis

Mitogenomic sequences from Brachyura species and two outgroup taxa (*Cherax destructor* and *Neopetrolisthes maculatus*) were retrieved from NCBI GenBank (accessions in Table 1). Protein-coding genes (13 PCGs) were aligned using MAFFT v7 under the invertebrate mitochondrial genetic code [24,25], with ambiguously aligned regions trimmed via Gblocks [26]. Consolidated alignments were generated using PhyloSuite’s sequence concatenation function [27,28]. Evolutionary model selection was optimized with PartitionFinder2 [29], followed by partitioned phylogenetic analyses using MrBayes v3.2 and IQ-TREE [30,31]. The GTR + I + G and MtRev + I + G + F models were chosen as the best models for nucleotide and amino acid phylogenetic analysis. The MrBayes software v3.2 underwent ten million generations with four chains, sampling every 100 generations, with a burn-in step of 5000 generations. The average standard deviation of the split frequency was less than 0.01, indicating convergence. The IQ-TREE software version 2 was run with 1000 bootstrapped replicates, and results were analyzed using Tracer v1.6. The effective sample size (ESS) value was over 200, showing convergence of the chains in the Markov chain Monte Carlo. Phylogenetic trees were visualized using the online tool Interactive Tree Of Life [32].

## 3. Results and Discussion

### 3.1. Mitogenome Organization and Nucleotide Composition

**Figure 1:**
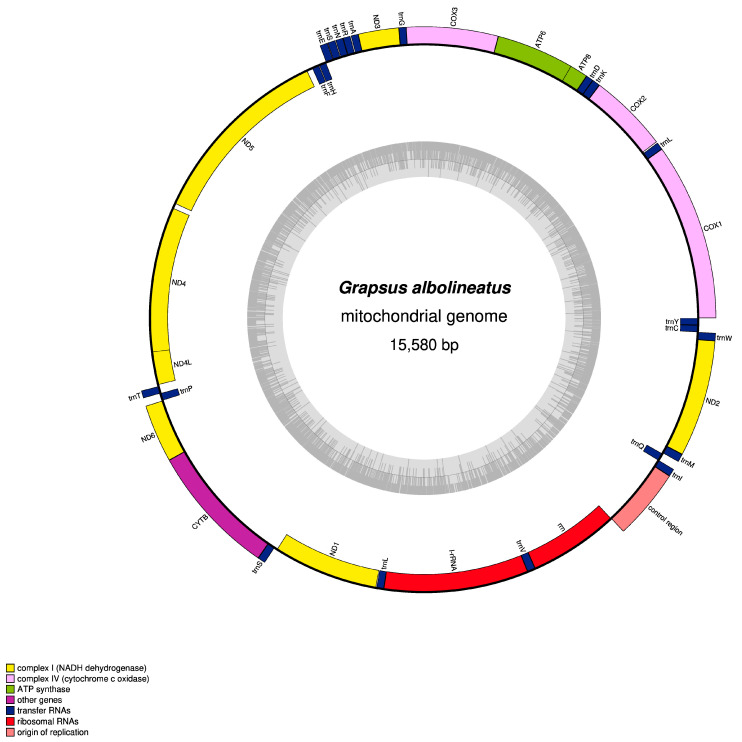
A circular map of the mitogenome of *Metopograpsus quadridentatus*. Protein-coding and ribosomal genes are presented with standard abbreviations. Transfer RNA (tRNA) genes are shown by single-letter abbreviations, except for S1 = AGN, S2 = UCN, L1 = CUN, and L2 = UUR. The thick lines outside the circle indicate the major strand, whereas those inside the circle indicate the minor strand.

**Figure 2:**
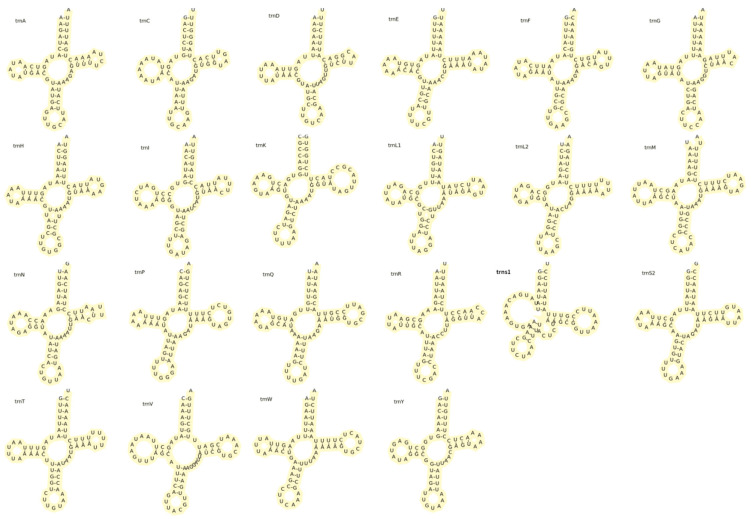
Putative secondary structure of transfer RNA (tRNA) genes of mitogenome of *Metopograpsus quadridentatus*.

The mitochondrial genome of *M. quadridentatus* exhibits a covalently closed circular configuration with double-stranded topology, spanning 15,523 base pairs (Figure 1). Consistent with typical Brachyuran mitochondrial architecture, it harbors 37 canonical genes: 13 protein-coding genes (PCGs), 22 transfer RNA (tRNA) genes, 2 ribosomal RNA (rRNA) genes, and a non-coding AT-rich regulatory region (Figure 2). Gene strand distribution reveals 14 tRNA genes (trnL2, trnK, trnD, trnG, trnA, trnR, trnN, trnS1, trnE, trnT, trnS2, trnI, trnM, and trnW) and all PCGs encoded on the minority strand, while the remaining genes (8 tRNAs, 2 rRNAs, and the AT-rich region) reside on the majority strand (Table 2). Genomic nucleotide composition demonstrates marked AT bias, with adenine (34.4%) and thymine (36.3%) collectively constituting 70.7% of the total bases, contrasted by cytosine (19.6%) and guanine (10.3%). The AT-rich region accounts for 70.4% of its sequence. Calculated compositional skews (AT skew = [A − T]/[A + T] = −0.025; GC skew = [G − C]/[G + C] = −0.314) indicate moderate adenine depletion and pronounced guanine deficiency. Comparative analysis across Brachyura reveals that *M. quadridentatus* shares the negative GC skew trend, while only 10 congeners exhibit marginally positive AT skew values (Table 3).

### 3.2. Protein-Coding Genes

**Figure 3:**
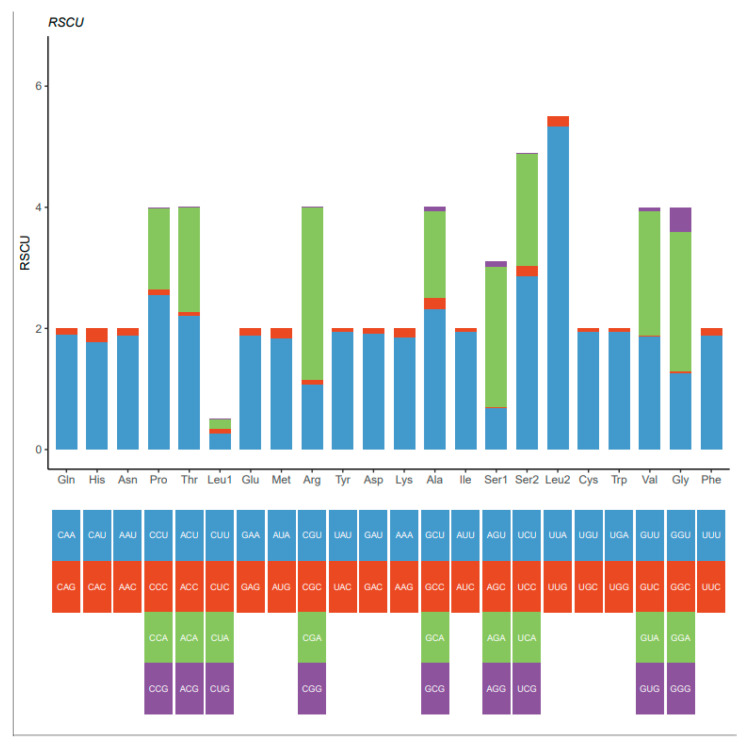
The relative synonymous codon usage (RSCU) values of the mitogenome of *Metopograpsus quadridentatus*.

As shown in Table 2, the 13 protein-coding genes (PCGs) varied in length, ranging from 159 bp (atp8) to 1734 bp (nad5). The PCG region of the mitochondrial genome of *M. quadridentatus* was 11,172 bp in length and consisted of 13 genes (nad1–6, nad4L, cox1–3, atp6, atp8, and cytb). The start codon for 12 of the 13 protein-coding genes is ATG (T/G), while the start codon for the nad4 gene is CTG. The mitochondrial genome of *M. quadridentatus* exhibits standard termination codons (TAA or serine-encoding TCG) in ten protein-coding genes (PCGs), whereas cox1, cox2, and cytb feature incomplete T-nucleotide stop signals (Table 2). Codon utilization patterns across PCGs are summarized in Table 3, revealing a total of 3724 codons. Leucine (Leu, 15.9%) predominates as the most abundant amino acid, followed by serine (Ser, 9.80%) and phenylalanine (Phe, 8.83%). The highest-frequency codons include UUA (Leu), UUU (Phe), and AUU (isoleucine) (Table 4). Relative synonymous codon usage (RSCU) profiles for *M. quadridentatus* (Figure 3) corroborate these findings, validating codon bias trends observed in *M. quadridentatus*.

### 3.3. Transfer RNA and Ribosomal RNA Genes and Control Region

The mitochondrial genome of *M. quadridentatus* includes two ribosomal RNA genes, *rrnL* (1323 bp) and *rrnS* (832 bp), with a *trnV* gene positioned between them—a conserved feature among metazoans [55]. The genome encodes 22 tRNA genes (Table 2), ranging from 63 to 74 nucleotides (total combined length: 1475 bp). Eight tRNAs (*trnH*, *trnF*, *trnP*, *trnL1*, *trnV*, *trnQ*, *trnC*, and *trnY*) were encoded on the minority strand. tRNAscan-SE analysis predicted canonical cloverleaf secondary structures for all tRNAs (Figure 2), except *trnS1*, which exhibited an elongated dihydroxyuridine (DHU) arm and a distinctive auxiliary loop, a structural anomaly consistent with Brachyuran mitogenomes [56]. Genomic organization analysis identified 18 nucleotide overlaps (1–32 bp) and five intergenic spacers (2–7 bp), excluding the control region. A 568 bp control region, positioned between *rrnS* and *trnI*, harbors conserved sequences associated with replication origin and transcriptional regulation (Table 2).

### 3.4. Gene Arrangement

**Figure 4:**
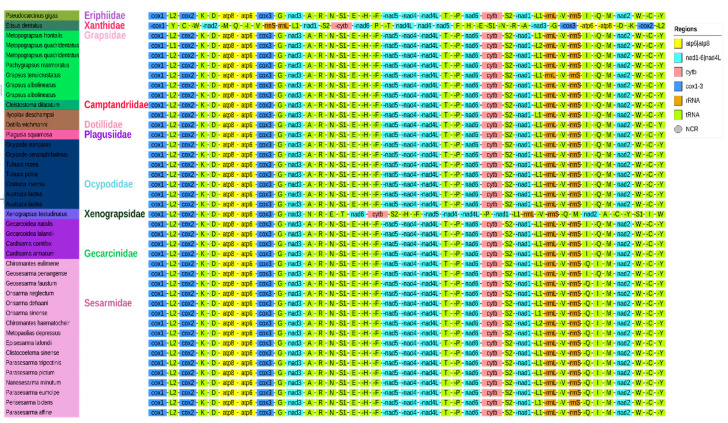
The gene order patterns of Brachyuran species used in this study.

Mitochondrial gene rearrangement is a valuable tool for studying the evolutionary relationships of organisms at higher taxonomic levels [57]. In recent years, this technique has been used to elucidate the evolutionary history of various groups such as birds, marsupials, echinoderms, nematodes, and others [58]. When comparing the gene order of the entire mitochondrial genome of *M. quadridentatus* to the Pancrustacean ground pattern, we observed that, except for the translocation of trnH, the gene sequences were identical (Figure 4). The mitochondrial genome of *Eucrate crenata* exhibits a notable rearrangement involving the translocation of trnH-cac, which deviates from the conserved gene order typically observed in decapods. In most brachyuran crabs, trnH-cac is positioned between nad5 and nad4, a pattern considered ancestral for the group. However, in E. crenata, this tRNA gene is relocated between trnE-gaa and trnF-ttc, marking a significant structural divergence [59]. Such rearrangements, though uncommon in Brachyura, are increasingly recognized as phylogenetically informative markers [19]. The trnH translocation observed here may reflect lineage-specific evolutionary dynamics, potentially arising from mechanisms such as tandem duplication–random loss (TDRL) or recombination-mediated processes [42]. Additionally, when compared to the gene rearrangement of other brachyura species in our study, we found that the gene order was the same among families Eriphiidae, Grapsidae, Camptandriidae, Dotillidae, Plagusiidae, Ocypodidae, and Gecarcinidae. However, in Sesamidae, gene rearrangement was observed in the transposition of trnQ and trnI. The mechanism of mitochondrial genome rearrangement is believed to involve errors in light-strand replication and gene tandem duplication, which can occur due to replication errors such as incorrect start or stop signals and slipped mispairing [60]. The gene order of Varunidae, Macrophthalmidae, and one Pinnotheridae species, Tritodynamia horvathi, are the same. The gene rearrangement pattern of Homolidae, Raninidae, Orithyiidae, Oregoniidae, Majidae, Bythograeidae, Oziidae, Xanthidae, Leucosiidae, Matutidae, Eriphiidae, Menippidae, Carpiliidae, Ovalipidae, Geryonidae, Portunidae, and some Potamidae species are the same or similar [19]. This rearrangement gains further significance when contextualized within the phylogenetic framework [43]. While gene order conservation is often emphasized in mitogenomic studies, the trnH translocation in Brachyura underscores the utility of structural variations in resolving deep phylogenetic nodes, particularly within Decapoda, where traditional morphological classification faces challenges [25]. Our study also revealed frequent gene rearrangements in tRNAs, protein-coding genes, and rRNAs in *Xenograpsus testudinatus* and *Etisus dentus*, suggesting the occurrence of mitochondrial gene recombination [54].

### 3.5. Phylogenetic Analysis

**Figure 5:**
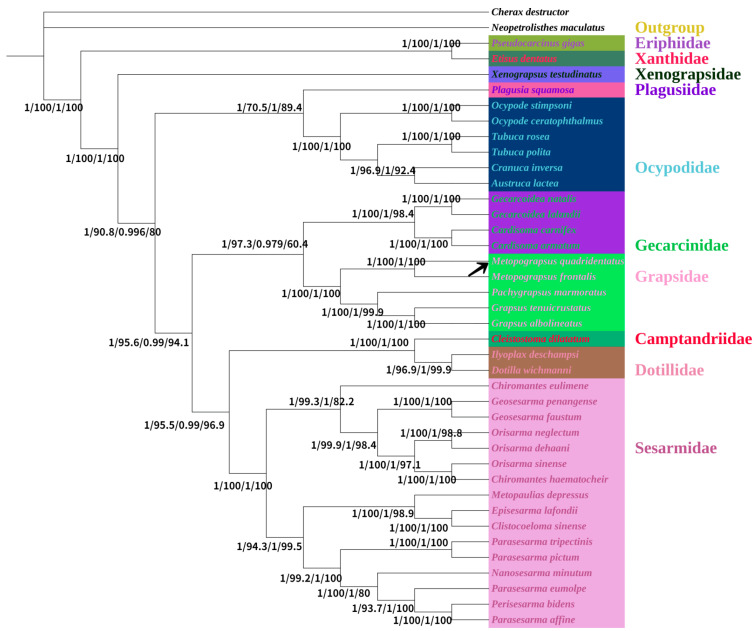
Phylogenetic tree inferred from nucleotide sequences and protein-coding genes of 13 protein-coding genes (PCGs) of mitogenome using Bayesian inference (BI) and maximum likelihood (ML) estimation. Numbers on nodes are BI posterior probability (nucleotide), ML bootstrap support (nucleotide), BI (amino acid), and ML (amino acid).

Phylogenetic reconstruction was initiated by identifying taxonomically proximate Brachyura species through NCBI BLAST homology screening. Nucleotide and amino acid sequences of 13 protein-coding genes (PCGs) from Brachyuran taxa (Table 1) were employed to resolve the phylogenetic position of *M. quadridentatus*. Topological congruence between the BI and ML trees permitted integration into a consensus phylogeny (Figure 5). Both analyses robustly placed *M. quadridentatus* and *M. frontalis* within a monophyletic clade, supported by statistically robust nodal values (posterior probability ≥ 0.95; bootstrap ≥ 90%), strongly suggesting a sister-group relationship between these congeners. Both *M. quadridentatus* and *M. frontalis* were clustered in the same branch with *Grapsidae* species. We can infer from this that *M. quadridentatus* belongs to genus *Metopograpsus*, family *Grapsidae*, which is consistent with the findings of a previous study [19]. The phylogenetic trees based on amino acid and nucleotide sequences have different topologies. In the phylogenetic trees based on nucleotide sequences, the topology is ((((((Sesermidae + Gecarcinidae) + Xenograpsidae) + (Ocypodidae + Plagusiidae)) + (Camptandriidae + Dotillidae)) + Grapsidae) + (Eriphiidae + Xanthidae)). The topology of the phylogenetic trees based on amino acid sequences is (((((Sesermidae + (Camptandriidae + Dotillidae)) + (Grapsidae + Gecarcinidae)) + (Ocypodidae + Plagusiidae)) + Xenograpsidae) + (Eriphiidae + Xanthidae)). This robust support for their placement within Grapsidae aligns with broader efforts to clarify brachyuran systematics, similarly resolving Grapsidae as a cohesive lineage using mitochondrial PCGs [40]. However, the conflicting higher-level topologies between nucleotide- and amino acid-based trees—such as Sesarmidae clustering with Gecarcinidae/Xenograpsidae in nucleotide trees versus Camptandriidae/Dotillidae in amino acid trees—underscore persistent challenges in reconstructing deep brachyuran relationships. These discrepancies echo Shen et al., who noted that mitochondrial sequence data, while valuable, often struggle to resolve rapid radiations or ancient divergences due to homoplasy and saturation, particularly in nucleotide alignments [52]. The polyphyly of traditional superfamilies like Grapsoidea and Ocypodoidea in our study further reflects the unresolved nature of brachyuran classification. For instance, the close association of Potamoidea (Parathelphusidae + Potamidae) with Thoracotremata, rather than their marine relatives in Heterotremata, parallels ecological transitions observed in freshwater crabs like Potamon [19]. Such anomalies suggest that adaptive radiations in novel niches (e.g., freshwater habitats) may obscure phylogenetic signals, as seen in Potamon’s origin in Western Asia and subsequent diversification across the Mediterranean [61]. This ecological divergence complicates molecular phylogenetics, as convergent adaptations can mimic shared ancestry, necessitating integrative approaches that combine mitochondrial data with nuclear loci or morphological traits [62]. The incongruent placements of Sesarmidae and Gecarcinidae in our trees also align with broader debates in brachyuran systematics. Zhang et al. similarly reported unstable relationships among Sesarmidae, Gecarcinidae, and Xanthidae in mitochondrial phylogenies, emphasizing the need for expanded taxon sampling [35]. Shen et al. further demonstrated that mitochondrial genomes alone may fail to resolve deep nodes without comprehensive representation of major lineages, a limitation exacerbated by the uneven sampling of families like Camptandriidae and Xenograpsidae in our study [52]. The reason for such discrepancies is that the classification of Brachyura crabs was not perfect, and more phylogenetic studies of Brachyura crabs are needed in the future. Owing to the ongoing development of DNA sequencing technology and bioinformatics, molecular technology can now be used to classify phylogeny [1,61]. The computing power of computers is increasing, which can be used to process molecular data. Using bioinformatic technology to analyze the mitogenome in the future will aid in evolutionary biology studies to classify crabs.

## 4. Conclusions

In this study, we analyzed the whole mitogenome of *M. quadridentatus*. Using gene arrangement patterns and phylogenetic analysis, we suggested that *M. quadridentatus* belongs to the Grapsidae family. While mitochondrial sequences remain a cornerstone of brachyuran phylogenetics, their limitations in resolving deep nodes call for methodological innovation. By combining mitochondrial data with nuclear markers, time-calibrated models, and robust taxon sampling, future studies can unravel the evolutionary history of Brachyura, particularly for ecologically divergent lineages like Grapsidae and Potamoidea.

## Figures and Tables

**Table 1:** List of Brachyuran species used in this study.

Accession No.	Species	Size (bp)	A + T%	Reference
MF198251.1	*Metopograpsus quadridentatus*	15,523	70.4	this study
OL661264.1	*Cleistostoma dilatatum*	15,443	69.0	[4]
NC_063602.1	*Plagusia squamosa*	15,460	69.9	unpulished
NC_063149.1	*Episesarma lafondii*	15,640	75.9	[5]
NC_061931.1	*Parasesarma eumolpe*	15,646	75.5	[19]
NC_057477.1	*Cardisoma armatum*	15,586	69.0	[19]
NC_057475.1	*Gecarcoidea lalandii*	15,575	75.0	[19]
NC_054248.1	*Etisus dentatus*	15,884	71.9	[33]
NC_051868.1	*Perisesarma bidens*	15,641	74.8	[34]
NC_047209.1	*Chiromantes eulimene*	15,894	75.5	[35]
NC_046797.1	*Ocypode stimpsoni*	15,557	67.8	[36]
NC_042401.1	*Austruca lactea*	15,659	69.4	unpublished
NC_042152.1	*Metopograpsus frontalis*	15,587	69.8	[37]
NC_042142.1	*Chiromantes haematocheir*	15,899	75.6	[38]
NC_041212.1	*Orisarma dehaani*	15,917	75.7	unpublished
NC_040977.1	*Nanosesarma minutum*	15,637	77.7	[39]
NC_039990.1	*Parasesarma affine*	15,638	74.8	[40]
NC_039111.1	*Cranuca inversa*	15,677	71.0	[41]
NC_039106.1	*Tubuca polita*	15,672	71.6	[41]
NC_039105.1	*Cardisoma carnifex*	15,597	68.8	[41]
NC_038180.1	*Dotilla wichmanni*	15,600	68.5	[42]
NC_038066.1	*Parasesarma pictum*	15,611	75.6	[43]
MZ725941.1	*Geosesarma penangense*	15,955	78.4	unpublished
MZ725940.1	*Geosesarma faustum*	15,880	78.4	[44]
MW255974.1	*Ocypode ceratophthalmus*	15,555	69.5	[45]
MN072632.2	*Tubuca rosea*	15,643	71.1	unpublished
MH816962.2	*Gecarcoidea natalis*	15,545	72.2	unpublished
MH796169.1	*Austruca lactea*	15,661	69.6	[46]
MF198247.1	*Grapsus albolineatus*	15,580	67.4	[47]
KX156954.1	*Orisarma neglectum*	15,920	75.6	unpublished
KX118277.1	*Metopaulias depressus*	15,765	77.3	[48]
KU589292.1	*Clistocoeloma sinense*	15,706	75.7	unpublished
KU343209.2	*Parasesarma tripectinis*	15,612	74.2	unpublished
KT878721.1	*Grapsus tenuicrustatus*	15,858	65.0	[49]
KR336554.1	*Orisarma sinense*	15,905	75.7	unpublished
KC107816.1	*Neopetrolisthes maculatus*	15,324	71.2	[50]
JF909979.1	*Ilyoplax deschampsi*	15,460	69.6	[51]
EU727203.1	*Xenograpsus testudinatus*	15,798	73.9	[52]
AY562127.1	*Pseudocarcinus gigas*	15,515	70.5	[53]
AY383557.2	*Cherax destructor*	15,894	62.4	[54]

**Table 2:** Annotation of complete mitochondrial genome of *Metopograpsus quadridentatus*.

Gene	From	To	Size	Intergenic Nucleotides	Start	Stop	Strand
*cox1*	1	1534	1534		ATG	T	H
*trnL1*	1535	1601	67				H
*cox2*	1610	2297	688	8	ATG	T	H
*trnK*	2298	2366	69				H
*trnD*	2367	2429	63				H
*atp8*	2430	2588	159		ATG	TAA	H
*atp6*	2582	3255	674	−7	ATT	TA	H
*cox3*	3256	4046	791		ATG	TA	H
*trnG*	4047	4112	66				H
*nad3*	4113	4463	351		ATT	TAA	H
*trnA*	4462	4525	64	−2			H
*trnR*	4528	4591	64	2			H
*trnN*	4600	4668	69	8			H
*trnS1*	4673	4740	68	4			H
*trnE*	4742	4810	69	1			H
*trnH*	4813	4877	65	2			L
*trnF*	4882	4944	63	4			L
*nad5*	4970	6703	1734	25	ATG	TAG	L
*nad4*	6736	8085	1350	32	GTG	TAA	L
*nad4L*	8079	8381	303	−7	ATG	TAA	L
*trnT*	8399	8464	66	17			H
*trnP*	8465	8531	67				L
*nad6*	8534	9039	506	2	ATT	TA	H
*cytb*	9040	10,174	1135		ATG	T	H
*trnS2*	10,175	10,241	67				H
*nad1*	10,260	11,207	948	18	ATG	TAA	L
*trnL2*	11,230	11,296	67	22			L
*rrnL*	11,297	12,619	1323				L
*trnV*	12,623	12,696	74	3			L
*rrnS*	12,698	13,529	832	1			L
*trnI*	14,098	14,166	69	568			H
*trnQ*	14,164	14,232	69	−3			L
*trnM*	14,240	14,309	70	7			H
*nad2*	14,313	15,320	1008	3	ATT	TAG	H
*trnW*	15,319	15,386	68	−2			H
*trnC*	15,390	15,452	63	3			L
*trnY*	15,456	15,523	68	3			L

**Table 3:** Codon number and relative synonymous codon usage (RSCU) in *Metopograpsus quadridentatus*. * represents the termination codon.

Codon	Count	RSCU	Codon	Count	RSCU	Codon	Count	RSCU	Codon	Count	RSCU
UUU(F)	264	1.6	UCU(S)	118	2.59	UAU(Y)	125	1.64	UGU(C)	33	1.69
UUC(F)	65	0.4	UCC(S)	24	0.53	UAC(Y)	27	0.36	UGC(C)	6	0.31
UUA(L)	311	3.15	UCA(S)	67	1.47	UAA(*)	5	1.43	UGA(W)	82	1.69
UUG(L)	64	0.65	UCG(S)	8	0.18	UAG(*)	2	0.57	UGG(W)	15	0.31
CUU(L)	110	1.11	CCU(P)	62	1.65	CAU(H)	46	1.08	CGU(R)	19	1.38
CUC(L)	25	0.25	CCC(P)	24	0.64	CAC(H)	39	0.92	CGC(R)	4	0.29
CUA(L)	73	0.74	CCA(P)	56	1.49	CAA(Q)	63	1.73	CGA(R)	27	1.96
CUG(L)	9	0.09	CCG(P)	8	0.21	CAG(Q)	10	0.27	CGG(R)	5	0.36
AUU(I)	258	1.73	ACU(T)	95	2.01	AAU(N)	97	1.4	AGU(S)	30	0.66
AUC(I)	41	0.27	ACC(T)	31	0.66	AAC(N)	42	0.6	AGC(S)	9	0.2
AUA(M)	173	1.61	ACA(T)	56	1.19	AAA(K)	76	1.69	AGA(S)	72	1.58
AUG(M)	42	0.39	ACG(T)	7	0.15	AAG(K)	14	0.31	AGG(S)	37	0.81
GUU(V)	115	1.71	GCU(A)	116	2.25	GAU(D)	43	1.25	GGU(G)	60	1.04
GUC(V)	8	0.12	GCC(A)	33	0.64	GAC(D)	26	0.75	GGC(G)	18	0.31
GUA(V)	121	1.8	GCA(A)	49	0.95	GAA(E)	55	1.49	GGA(G)	108	1.88
GUG(V)	25	0.37	GCG(A)	8	0.16	GAG(E)	19	0.51	GGG(G)	44	0.77

**Table 4:** Nucleotide composition and skewness of mitochondrial genome.

Species	Family	Size (bp)	A (%)	T (%)	C (%)	G (%)	AT Skew
*Metopograpsus frontalis*	*Grapsidae*	15,587	32.8	37	19.3	11	−0.06
*Orisarma dehaani*	*Sesarmidae*	15,917	37.5	38.2	14.8	9.5	−0.01
*Orisarma neglectum*	*Sesarmidae*	15,920	37.4	38.2	14.9	9.5	−0.01
*Orisarma sinense*	*Sesarmidae*	15,905	37.4	38.3	14.9	9.4	−0.012
*Chiromantes eulimene*	*Sesarmidae*	15,894	37.1	38.4	14.8	9.7	−0.017
*Episesarma lafondii*	*Sesarmidae*	15,640	37	38.9	14.7	9.4	−0.025
*Chiromantes haematocheir*	*Sesarmidae*	15,899	37.3	38.3	15	9.4	−0.013
*Clistocoeloma sinense*	*Sesarmidae*	15,706	37.1	38.6	14.9	9.4	−0.02
*Geosesarma penangense*	*Sesarmidae*	15,955	38.3	40.1	13.1	8.5	−0.023
*Cardisoma armatum*	*Gecarcinidae*	15,586	35.3	33.7	20.7	10.3	0.023
*Perisesarma bidens*	*Sesarmidae*	15,641	36.6	38.2	15.1	10.1	−0.021
*Cardisoma carnifex*	*Gecarcinidae*	15,597	35.4	33.4	21	10.1	0.028
*Parasesarma eumolpe*	*Sesarmidae*	15,646	36.7	38.8	14.7	9.8	−0.027
*Parasesarma tripectinis*	*Sesarmidae*	15,612	36.2	38	15.7	10.1	−0.024
*Nanosesarma minutum*	*Sesarmidae*	15,637	38	39.7	13.4	8.9	−0.022
*Geosesarma faustum*	*Sesarmidae*	15,880	38.7	39.7	13.2	8.3	−0.013
*Metopaulias depressus*	*Sesarmidae*	15,765	37.9	39.4	14	8.7	−0.019
*Plagusia squamosa*	*Plagusiidae*	15,460	34.3	35.6	18.9	11.2	−0.018
*Cleistostoma dilatatum*	*Camptandriidae*	15,443	34.3	34.7	19.5	11.4	−0.006
*Grapsus albolineatus*	*Grapsidae*	15,580	33.4	34	20.5	12.1	−0.01
*Cranuca inversa*	*Ocypodidae*	15,677	35.8	35.2	18.1	10.9	0.008
*Dotilla wichmanni*	*Dotillidae*	15,600	33.8	34.7	20.7	10.8	−0.014
*Ilyoplax deschampsi*	*Dotillidae*	15,460	34.1	35.5	19.7	10.7	−0.019
*Austruca lactea*	*Ocypodidae*	15,659	34.8	34.6	18.5	12	0.003
*Ocypode stimpsoni*	*Ocypodidae*	15,557	33.7	34.1	20.8	11.4	−0.006
*Ocypode ceratophthalmus*	*Ocypodidae*	15,555	33.8	35.7	19.4	11.1	−0.028
*Pseudocarcinus gigas*	*Eriphiidae*	15,515	35	35.5	18.7	10.8	−0.006
*Gecarcoidea lalandii*	*Gecarcinidae*	15,575	37.7	37.3	15.8	9.2	0.005
*Gecarcoidea natalis*	*Gecarcinidae*	15,545	36	36.2	15.1	9.1	−0.003
*Parasesarma pictum*	*Sesarmidae*	15,611	36.6	39	14.6	9.8	−0.032
*Tubuca rosea*	*Ocypodidae*	15,643	36.6	34.5	18.6	10.3	0.029
*Parasesarma affine*	*Sesarmidae*	15,638	36.6	38.2	15.1	10.1	−0.022
*Tubuca polita*	*Ocypodidae*	15,672	36.6	35	17.5	10.9	0.022
*Austruca lactea*	*Ocypodidae*	15,661	34.9	34.7	18.5	12	0.003
*Etisus dentatus*	*Xanthidae*	15,884	37.9	34	10.4	17.8	0.054
*Grapsus tenuicrustatus*	*Grapsidae*	15,858	31.9	33.1	22.8	12.1	−0.018
*Xenograpsus testudinatus*	*Xenograpsidae*	15,798	36.7	37.2	16.8	9.3	−0.007
*Neopetrolisthes maculatus*	*Porcellanidae*	15,324	34.9	36.3	17.4	11.4	−0.02
*Cherax destructor*	*Parastacidae*	15,894	32.1	30.3	24.1	13.5	0.029
*Metopograpsus quadridentatus*	*Grapsidae*	15,523	34.3	36.1	19.5	10.2	−0.025

## Data Availability

The datasets generated for this study can be found in GenBank with accession no. MF198251.
